# Skin Lesions in European Hibernating Bats Associated with *Geomyces destructans*, the Etiologic Agent of White-Nose Syndrome

**DOI:** 10.1371/journal.pone.0074105

**Published:** 2013-09-04

**Authors:** Gudrun Wibbelt, Sébastien J. Puechmaille, Bernd Ohlendorf, Kristin Mühldorfer, Thijs Bosch, Tamás Görföl, Karsten Passior, Andreas Kurth, Daniel Lacremans, Frédéric Forget

**Affiliations:** 1 Leibniz Institute for Zoo and Wildlife Research, Berlin, Germany; 2 School of Biology and Environmental Science, University College Dublin, Dublin, Ireland; 3 Sensory Ecology Group, Max Planck Institute for Ornithology, Seewiesen, Germany; 4 Zoological Institute & Museum, Greifswald University, 17489 Greifswald, Germany; 5 Federal Office for Bat Protection Saxony-Anhalt, Roßla, Germany; 6 Resource Ecology Group, Wageningen University, Wageningen, The Netherlands; 7 Institute for Biology, Faculty of Veterinary Science, Szent István University, Budapest, Hungary; 8 Department of Zoology, Hungarian Natural History Museum, Budapest, Hungary; 9 Nature and Biodiversity Conservation Union Southern Lower-Saxony, Nordstemmen, Germany; 10 Robert Koch Institute, Berlin, Germany; 11 Institut de Pathogénétique, Gosselies, Belgium; 12 Natagora, Plecotus Working Group, Namur, Belgium; California Department of Public Health, United States of America

## Abstract

White-nose syndrome (WNS) has claimed the lives of millions of hibernating insectivorous bats in North America. Its etiologic agent, the psychrophilic fungus *Geomyces destructans*, causes skin lesions that are the hallmark of the disease. The fungal infection is characterized by a white powdery growth on muzzle, ears and wing membranes. While WNS may threaten some species of North American bats with regional extinction, infection in hibernating bats in Europe seems not to be associated with significant mortality. We performed histopathological investigations on biopsy samples of 11 hibernating European bats, originating from 4 different countries, colonized by *G. destructans*. One additional bat was euthanized to allow thorough examination of multiple strips of its wing membranes. Molecular analyses of touch imprints, swabs and skin samples confirmed that fungal structures were *G. destructans*. Additionally, archived field notes on hibernacula monitoring data in the Harz Mountains, Germany, over an 11-year period (2000–2011) revealed multiple capture-recapture events of 8 banded bats repeatedly displaying characteristic fungal colonization. Skin lesions of *G. destructans*-affected hibernating European bats are intriguingly similar to the epidermal lesions described in North American bats. Nevertheless, deep invasion of fungal hyphae into the dermal connective tissue with resulting ulceration like in North American bats was not observed in the biopsy samples of European bats; all lesions found were restricted to the layers of the epidermis and its adnexae. Two bats had mild epidermal cupping erosions as described for North American bats. The possible mechanisms for any difference in outcomes of *G. destructans* infection in European and North American bats still need to be elucidated.

## Introduction

Since 2006 North America has experienced mass mortalities of estimated more than 6.7 million hibernating bats [1] caused by the cold-loving keratinophilic fungus, *Geomyces destructans* [2], [3]. Epidermal fungal colonization of affected bats is characterized by distinct white fungal patches on snout, ears and wing membranes [4]–[6]; the facial distribution of the fungus lead to the name of “white-nose syndrome” (WNS). Signs of WNS in affected hibernacula include aberrant hibernation behavior (day flight) and increased mortality rates, whereas to confirm the diagnosis of WNS the identification of typical histopathologic lesions and genetic identification of the fungus is required [1], [6], [7]. Since its first detection in New York state the fungal infection has spread through hibernacula in most neighboring states along the east coast. It has also moved further west, to Tennessee and Oklahoma, as well as crossed the Canadian border into Ontario, Quebec and Nova Scotia [8]. Seven bat species are currently known to be affected by WNS, but it is feared that more species will become involved since the habitat range of additional hibernating bat species is located in western areas. As a result of WNS, many North American hibernacula that previously contained thousands to hundreds of thousands of bats have been decimated [9]. Population viability analyses predict that *Myotis lucifugus* (the little brown bat), formerly one of the most abundant bat species in many regions of North America, could become extinct in 16–20 years [9]. The impact of WNS on biodiversity and on agricultural economy remains unknown, but will most likely be of major significance [10].

Fungal infectious agents can either serve as a primary pathogen or they may invade secondary to predisposing factors like co-infections by other pathogens. Bats dying of WNS had no consistent significant pathologic changes in their internal organs neither bacteriological nor virological analyses revealed consistent evidence for any known pathogen [4], yet experimental infections with *G. destructans* resulted in the same lesions that occur in natural infections [2], [3]. Many of the dead bats are found in an emaciated state which is thought to be due to the noted increased frequency of arousal cycles in affected bats, resulting in premature consumption of fat reserves [3], [11]. A recent study of naturally infected *M. lucifigus* supports this hypothesis, as WNS affected bats showed decreased torpor bout duration [7]. Furthermore, wing membrane lesions associated with WNS are hypothesized to cause an imbalance of homeostasis of body fluids ultimately leading to the death of the affected animals [11].

Dermatophyte infections are restricted to superficial skin structures, i.e. corneal stratum or hair cuticle. In contrast, infections by *G. destructans* in North American bats range from cup-like intraepidermal colonies with erosions to severe ulceration of the affected skin and deep invasion by fungal hyphae into the underlying dermal connective tissue. However, even extensive *G. destructans* invasion lacks noticeable cellular inflammatory response in its early stages [6]. As hibernation has been shown to reduce body functions, including the immune system to a minimal level in other hibernators [12], it is assumed that the lack of inflammation against invading fungal hyphae reflects this temporary physiologic unresponsiveness in bats.

In Europe, a wide distribution of *G. destructans* was found in 13 European countries involving 7 different species of hibernating bats [13]. Despite a long standing tradition of annual hibernacula census in a number of European countries, no fungus-associated mass mortalities have been recorded. Furthermore, bats which were notably colonized with the fungus left their hibernacula uneventfully in spring alongside their unaffected colony members [13]. Growth requirements, morphologic characteristics and the 100% sequence similarity within the ITS and SSU rRNA gene segments indicate that European *G. destructans* isolates are closely related to the US type strain [13]–[16].

In contrast to the management of bat populations in North America, all bat species in Europe are protected under the European Union’s 1992 Conservation of Wild Flora and Fauna Directive (http://ec.europa.eu/environment/nature/legislation/habitatsdirective/index_en.htm) (92/43/EEC) and the Agreement on the Conservation of Populations of European Bats (www.eurobats.org) both of which prohibit invasive sampling of bats. Owing to these legislations, until recently it was not possible in most European countries to investigate fungal colonization of hibernating bats beyond touch imprints of fungal structures from the surface of the skin. With the emergence of WNS and the pressing need to shed light on the question of whether hibernating bats from Europe also had inflammatory reactions to *G. destructans*, governmental authorities granted limited permission for invasive sampling such as wing punch biopsies. Here, we describe histopathologic investigations of wing punch biopsies from bat skin colonized by *G. destructans* as well as long term field observations on capture-recapture events in hibernating bats with fungal growth.

## Materials and Methods

### Permits

For this study, exemption permits for the collection of punch biopsies of wing membranes from hibernating bats were given to designated bat activists registered for annual hibernacula monitoring, by the responsible regional governmental authorities (General Directorate for Agriculture, Natural Resources and Environment, Namur, Belgium (F. Forget), Préfecture du Cher, France (L. Arthur), Hanover region nature conservation agency, Hanover, Germany (K. Passior), Saxony-Anhalt Ministry for Agriculture and Environment, Magdeburg, Germany (B. Ohlendorf), Hungarian National Inspectorate for Environment, Nature and Water (T. Görföl)). Further permission under the Flora and Fauna Act was given to P. Lina of the Netherlands Centre for Biodiversity ‘Naturalis’ by the Netherlands Ministry of Economic Affairs, Agriculture and Innovation (FF/75/2003/169b, valid 5^th^ February 2007 through 12^th^ April 2010) to euthanize a single bat with visible fungal growth.

### Field Sampling

The aim of the study was to investigate the pathogenic effect of *G. destructans* colonizing the skin of hibernating European bats. Bat populations in most hibernacula in Europe are significantly smaller than those in North America. The average number of individual bats in the hibernacula in this study ranged from 20 to 200. So if *G. destructans* colonization were to be found in hibernacula, it is usually limited to one or a few affected animals. Of these bats only the most visibly severe cases of fungal growth were chosen for sampling in this study. Sampling dates were set towards the end of the hibernation period when the first bats of the respective colonies had already left the hibernacula. In this way sampled animals, unavoidably aroused during the handling process, would be minimally affected by the procedure. Body condition was estimated by the palpable thickness of subcutaneous brown fat tissue dorsal between the shoulders. Prior to biopsy sampling, adhesive tape touch imprints were taken for the German and Hungarian bats for microscopic fungal spore identification while swab samples were taken from the bat from France. A total of 10 live hibernating *M. myotis* with gross evidence of extensive fungal colonization on snout, ears and wing membranes were sampled by wing punch biopsies (see Table 1) including one bat from Belgium (BE 1), one bat from France (FR 1), 7 bats from Germany (GE 1–7) and one bat from Hungary (HU 1). Punch biopsies of 3 mm in diameter were taken at the border region of visible fungal growth and adjacent tissue and were stored in 70% ethanol. The Belgium sample (BE 1) was submitted to the Institut de Pathogénétique, Gosselies, Belgium. The French, German and Hungarian biopsies (FR 1, GE 1–7, HU 1) were sent to the Leibniz Institute for Zoo and Wildlife Research, Berlin, Germany. Wing punch biopsies are a widely accepted standard method [17] and routinely used in bat biology research for retrieving DNA samples for molecular analyses [18]. Resulting perforations of a similar size to those in our study have been shown to heal within a 1 to 4 weeks [19].

**Table 1:** Details on bat samples regarding bat species, origin, collection date, sampled tissue, histology result and accession numbers for ITS gene sequences.

Bat species	Country	Location	Collection date	Sample	Sample ID	Histology result	GenBank Acc. No.
*M. myotis*	Belgium	Wallonia	22/07/2011	W	BE 1	Gd restricted to str. corneum	n.a.
*M. myotis*	France	Cher	15/02/2012	W+uro	FR 1	Gd restricted to str. corneum	n.a.*
*M. myotis*	Germany	Lower Saxony	21/03/2011	W	GE 1	Gd in str. corneum+single cupping erosion filled withGd hyphae	JQ342818
*M. myotis*	Germany	Lower Saxony	21/03/2011	W	GE 2	Gd restricted to str. corneum	JQ342819
*M. myotis*	Germany	Saxony-Anhalt	29/03/2011	W	GE 3	Gd restricted to str. corneum	JQ342820
*M. myotis*	Germany	Saxony- Anhalt	21/03/2011	W	GE 4	Gd restricted to str. corneum	JQ342821
*M. myotis*	Germany	Saxony- Anhalt	22/03/2011	W	GE 5	Gd in str. corneum+hair shaft encased by Gd hyphae	JQ342822
*M. myotis*	Germany	Saxony- Anhalt	21/03/2011	W	GE 6	Gd restricted to str. corneum	JQ342823
*M. myotis*	Germany	Saxony- Anhalt	22/03/2011	W	GE 7	Gd restricted to str. corneum+aerial hyphae	JQ342824
*M. myotis*	Hungary	Kislőd	24/03/2010	W+uro	HU 1	Gd in str. corneum+intraepidermal microabscesseswith Gd hyphae	JF502405
*M. daubentonii*	The Netherlands	Gelderland	09/03/2010	Euthanized bat	NL 1	Wing: Very few cupping erosions+intraepidermalpustules with Gd hyphae; snout: some hair folliclesfilled with Gd hyphae+Gd hyphae in mucosalepithelium of nasal orifice	JF502411

Samples refer to biopsy punches from wing or uropatagial membranes. One bat from the Netherlands was euthanized and multiple strips of wing membrane were investigated. If touch imprints or tissue samples, corresponding to the histological examined sites, were taken for molecular analysis, GenBank accession numbers (Acc. No.) are included; W = wing; Urop = uropatagium; Gd = *Geomyces destructans*; str. = stratum; n.a. = not applicable; * = sequence obtained was 100% identical to all other ITS sequences of this study.

Further, a single *M. daubentonii* from the Netherlands was euthanized for this study, which had multifocal fungal colonization on its wing membranes, its ears and around its snout. Euthanasia was performed by cervical dislocation in accordance to the guidelines for euthanasia of small mammals for technically skilled personnel [18], [20]. The carcass of the euthanized *M. daubentonii* (NL 1) was immediately frozen after death at –20°C for transport to allow subsequent molecular biology methods as well as histopathology.

**Figure 1:**
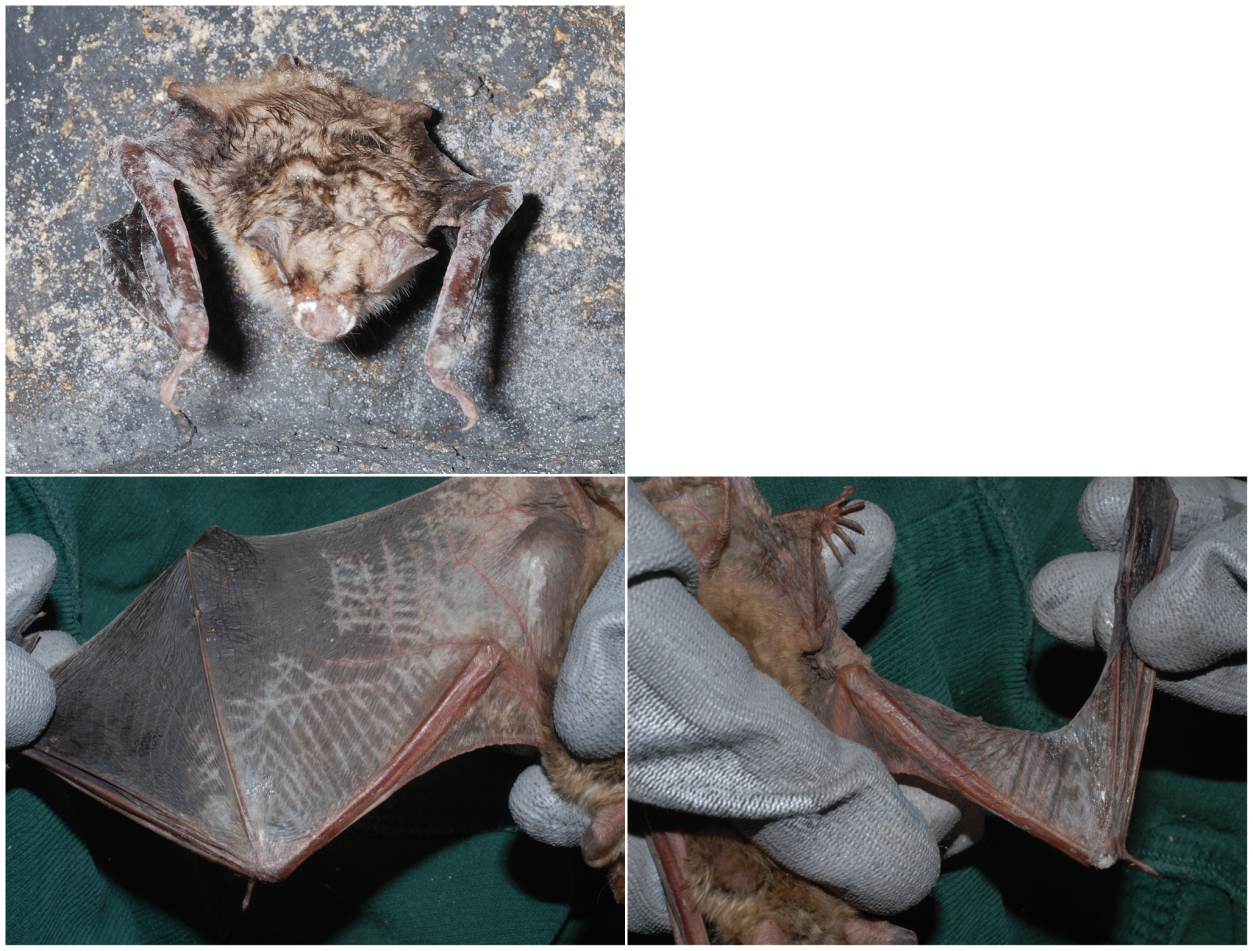
Emaciated *Myotis myotis* from a hibernaculum in France covered by *Geomyces destructans*. 1A: Old laceration of the left wing identified on the day of collection. 1B: Bat with improved body condition after some weeks of rehabilitation. 1C+D: Wing membranes after cleaning with notable depigmentation.

On February 15, 2012, the French *M. myotis* was detected in its hibernacula in poor body condition. As the left wing membrane was extensively torn, it can be assumed its pre-hibernation hunting success must have been markedly impaired, resulting in an insufficient fat storage for hibernation. Of all bats sampled this animal had the most visible evidence of fungal growth (Fig. 1). It was taken into a rehabilitation center to be sustained throughout the winter. Immediately upon arriving at the center a swab sample and a wing punch biopsy (Fig. 1A) were taken from one of the most affected areas.

### Molecular-biology Investigations

All adhesive tape or swab samples (no sample from the Belgian bat (BE 1) was collected for genetic analysis), as well as tissue samples of wing membrane, muzzle and ear of the Dutch *M. daubentonii* were investigated by PCR amplification of the fungal rRNA gene internal transcribed spacer (ITS) region DNA (ITS1, 5.8S, and ITS2). Total nucleic acids were extracted from all samples using PrepMan Ultra reagent (Applied Biosystems, Darmstadt, Germany) following the manufacturer’s instructions. Ribosomal RNA gene ITS region DNA was PCR amplified using primers ITS4 and ITS5 [21] and GoTaq DNA polymerase (Promega, Madison, Wisconsin). Cycling parameters were an initial 2 min denaturation at 98°C followed by 30 cycles of denaturation at 98°C for 10 s, annealing at 50°C for 30 s, and extension at 72°C for 1 min, with a final extension at 72°C for 7 min. All PCR products were further processed for direct sequencing and retrieved sequences were blasted against sequences of *G. destructans* in GenBank. Samples with 100% sequence similarity to the respective gene segments of the *G. destructans* type isolate NWHC 20631–21 (GenBank accession no. EU884921) were considered positive for *G. destructans* and their sequences were submitted to GenBank.

### Histopathology Investigations

The skin of the entire muzzle of the euthanized Dutch *M. daubentonii* (NL 1) was removed and dissected in cross sectional planes vertical to the longitudinal axis of the skull. Numerous long strips of the wing membranes, particularly between the fourth and fifth fingers, were cut and fixed in formalin for histology investigations.

Punch biopsies of the 10 live bats were examined with a dissection microscope to identify the site of fungal growth. Upon detection of fungal colonization, biopsies were cut diagonally to ensure that the maximum length of an affected wing membrane biopsy was sectioned for histological examination. Biopsy halves as well as the skin samples of muzzle, ear and wing membranes of the Dutch *M. daubentonii* were subsequently processed for histology investigations, embedded in liquid paraffin and serially sectioned at 3 µm. All sample material was completely used for these sequential sections and slides were stained alternating with hematoxylin-eosin and periodic acid Schiff stain.

### Banding and Recapture

Hibernacula located in the southeastern Harz Mountains, Germany, are annually monitored and census data, i.e. number of bats present, bat species, gender and banding numbers as well as unusual observations are recorded. Unmarked bats receive a new banding ring. All capture-recapture data were archived in field books and also submitted to the Dresden Bat Banding Center, Germany, to be included in a general banding database. Field books from 2000 onwards were searched for bat capture-recapture entries in connection with noted fungus colonization on live bats. Additionally, during the annual hibernacula census in Lengefeld, Saxony, Germany, 2 banded *M. myotis* (A45309, male; A45392, female) were noticed with fungal growth on the 5^th^ February 2011. Adhesive tape samples and photographs were taken. Three weeks later, on the 26^th^ February 2011, the same animals were re-observed and new photographs taken (E.& R. Francke).

## Results

All sampled bats, except FR 1, had the appearance of a healthy animal with round body shape, fluffy fur coat and moderate amounts of palpable subcutaneous brown fat tissue between their shoulders and were therefore considered to be of good to moderate body condition. Only FR 1 was of poor appearance with a very thin body outline and no palpable brown fat tissue and it was therefore classified emaciated (Fig. 1).

### Molecular Investigations and Dissection Microscopy

**Figure 2:**
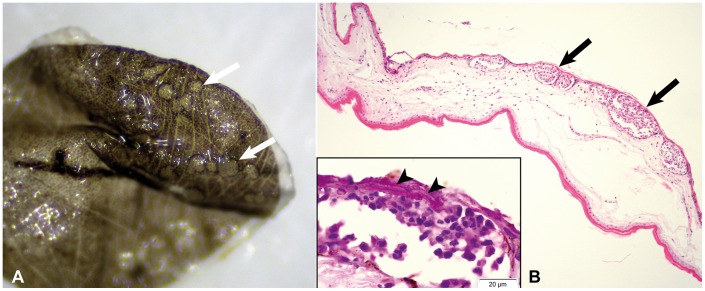
Biopsy of uropatagial membrane of *Myotis myotis* with acute inflammatory response against *Geomyces destructans*. 2A (sample HU 1): Dissection microscope image of punch biopsy with distinct circular lesions (arrows), where *G. destructans* had sloughed off during preparation. 2B: Histological cross section of 2A with multiple well-demarcated intraepidermal microabscesses (arrows). Hematoxylin-eosin staining. Inset: Hyphae of *G. destructans* invading the microabscesses (arrow heads). PAS staining.

Samples from a total of 11 bats from 4 countries were investigated (see Table 1). All samples, except the sample from Belgium (which was not analyzed genetically), had an ITS sequence 100% identical to Gd type isolate (GenBank accession no. EU884921). The hibernating *M. myotis* from Hungary was heavily colonized by fungus on its snout, ears, forearms, wing membranes, and uropatagium. The latter was biopsied at the site of superficial fine strands of white fungal growth. Upon arrival to the lab, the ethanol fixed uropatagium sample (HU 1) still had the fungal strands attached, but unfortunately sloughed off when the biopsy was removed from the vial a second time for photography. The fungal strands were associated with minute, well demarcated, circular, light grey lesions visible by dissection microscopy (Fig. 2A). In contrast to the Hungarian biopsy the wing punches from the 7 *M. myotis* from Germany (GE 1–7) did not reveal distinct lesions by low power dissection microscopy examination. Similarly, the dissection microscopy examination of the *M. daubentonii* carcass from the Netherlands did not reveal any evidence of fungal growth. The Belgian wing punch biopsy (BE 1) was not examined by dissection microscopy.

### Histopathologic Examination

**Figure 3:**
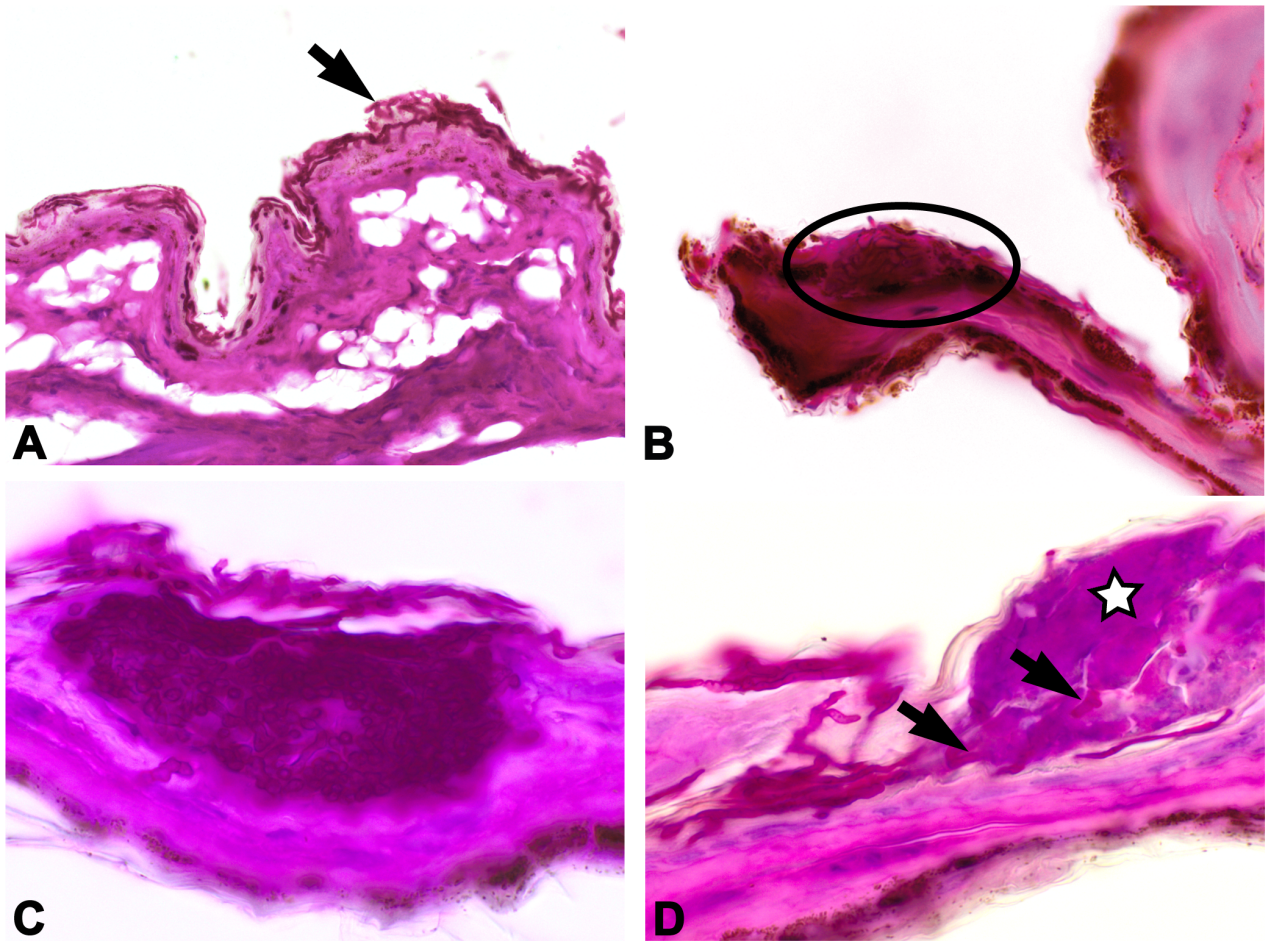
*Geomyces destructans* colonization of superficial epidermis occasionally associated with epidermal pustules. 3A: A representative wing biopsy punch (GE 7). 3B (wing punch GE 1): Small epidermal cluster of *G. destructans* hyphae (encircled). 3C (sample NL 1): Wing membrane of euthanized bat with cupping erosion and densely packed hyphae of *G. destructans* restricted to the epidermis. 3D (sample NL 1): Fungal hyphae (arrows) invasion into intraepidermal pustules (star). PAS staining.

All wing punch biopsies from the Belgian (BE 1) and 7 German bats (GE 1–7) contained fungal hyphae superficially located in the stratum corneum of the epidermis (Fig. 3A). In one biopsy (GE 1), a small focus of densely interwoven fungal hyphae, similar to the described intradermal cup-shaped colonies in North American bats [6], was located within the epidermal layers, but without dermal invasion (Fig. 3B). Another biopsy (GE 6) contained a hair follicle with intraluminal colonization by fungal hyphae growing in perpendicular orientation to the hair shaft and extending into the neighboring sebaceous gland. None of these fungal hyphae were associated with cellular inflammatory response. In contrast, the Hungarian biopsy of the uropatagium (HU 1) contained multiple intraepidermal microabscesses corresponding to the light grey circular discolorations visible via dissection microscopy. Microabscesses consisted of a mixed inflammatory cell population dominated by neutrophils (Fig. 2B). The covering epidermis was intact but colonized by fungal hyphae, which reached further into the underlying abscessations (Fig. 2B, inset). The adjacent dermis was moderately edematous with mild infiltration by neutrophils and plasma cells.

**Figure 4:**
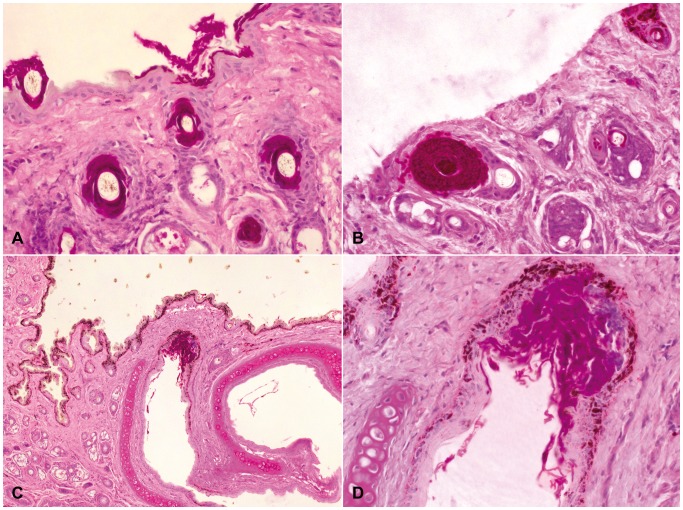
Skin of the muzzle of a *Geomyces destructans* infected *Myotis daubentonii* (sample NL 1). 4A: Multiple hair follicles with intraluminal colonization of *G. destructans* hyphae. 4B: Wall of a hair follicle replaced by marked growth of *G. destructans* hyphae. 4C+D: Cutaneous epithelium of one nare severely colonized by *G. destructans* with mild localized suppurative inflammation limited to the epithelial layer. PAS staining.

Examination of the euthanized Dutch *M. daubentonii* (NL 1) revealed numerous hair follicles of the skin of the snout densely filled with fungal hyphae. Occasionally the entire follicular epidermal isthmus was replaced by hyphae, but there was no inflammatory reaction (Fig. 4A+B). Similar to the wing biopsy punches described above, there was multifocal superficial colonization of fungal hyphae in the stratum corneum. Hyphae also extended above the haired skin bearing asymmetrical curved conidia characteristic for *G. destructans* [5] and corresponding with the macroscopically visible fungal growth. One of the nares had intraluminal fungal colonization of its dorsal cutaneous epithelium similar to the cupping erosions of the wing membranes. Here, fungal hyphae reached the bordering basement membrane and invoked a localized suppurative inflammation limited to the epithelial layer. There was no evidence for cellular response in the underlying connective tissue (Fig. 4C+D). Investigation of the euthanized *M. daubentonii* wing membranes showed abundant superficial colonization with fungal hyphae limited to the stratum corneum, but extending above the surface to create a dense network of aerial mycelium. There was one focal cluster of cup-shaped intradermal fungal hyphae in the wing membrane of this bat (Fig. 3C). Occasionally, remnants of asymmetrical curved conidia were detected amongst these fungal hyphae. To a lesser extent, multiple intracorneal and intraepidermal pustules were noted, which were associated with invading fungal hyphae (Fig. 3D). Interestingly, despite the marked neutrophilic accumulation within the microabscesses no further inflammatory cells were found in the adjacent dermis, even if abscesses and hyphae bulged into the deeper tissue. All internal organs of this bat were unremarkable and without evidence of disease.

Similar findings to the other biopsies were encountered in the uropatagial biopsy of the French bat (FR 1), which had marked superficial fungal growths with focally numerous hyphae intermingled with each other. Additionally, formation of superficial suppurative pustules was detected within the outer layer of the epidermal corneal stratum containing neutrophilic granulocytes and being covered by fungal hyphae. There was neither indication of deep tissue invasion by the fungus nor ulceration. The macroscopic images of the French bat (Fig. 1) show the extent of fungal colonization and depigmentation of the wing. The delineated crisscross pattern of the elastic wing membranes could suggest that these edges, as the most superficially located areas when the wings are folded up, are sites most easily covered by fungal spores.

### Recapture Data

Archived bat banding and census data books from 2000 to 2011 contained notes on 8 *M. myotis* (4 males, 4 females) banded and recaptured in subsequent years and displaying fungal growth, particularly around their muzzle, at different times of detection (Table 2). The longest capture-recapture entry of a single individual covers 11 years. Four bats were found twice in subsequent years with fungal growth. A further 4 animals were found once with fungus; one of these was recaptured 3 winters later without fungus, another one was found in a maternity colony 3 months later. During this period of 11 years, the bats remained philopatric to their chosen hibernaculum, except one individual.

**Table 2:** Retrospective data on capture/recapture of banded *Myotis myotis* with visible fungal colonization.

Banding-ID	Sex	Capture/recapture date	Hibernaculum (location, gallery)	Fungus
A 17581	f	21.02.2000	Rübeland, “Trinkwasserstollen”	no
		**08.03.2005**	same place	**yes**
		31.08.2008	same place	no
A 17586	f	21.02.2000	Rübeland, “Trinkwasserstollen”	no
		**08.03.2005**	same place	**yes**
A 21750	m	24.02.2000	Tresburg, “Luppbodestollen”	no
		06.02.2001	same place	no
		**31.03.2010**	same place	**yes**
		**30.03.2011**	same place	**yes**
A 22024	f	**17.02.2000**	Elbingerode, “Augustenstollen”	**yes**
		18.01.2001	same place	no
		06.03.2002	Elbingerode, “Pinge Charlotte”	no
		**18.01.2006**	same place	**yes**
A 22209	m	09.03.2000	Elbingerode, “Büchenbergstollen”	no
		**12.03.2001**	same place	**yes**
		28.12.2001	same place	no
		**02.03.2002**	same place	**yes**
		30.12.2009	same place	no
		22.03.2011	same place	no
A 22213	m	**09.03.2000**	Elbingerode, “Büchenbergstollen”	**yes**
		**12.03 2001**	same place	**yes**
		28.12.2001	same place	no
A 88088	m	15.01.2009	Silberhütte, “Fürst-Viktor-Stollen”	no
		**29.03.2011**	same place	**yes**
A 93149	f	**29.03.2011**	Treseburg, “Falkenklippenstollen”	**yes**
		12.06.2011	Bleicherode (maternity colony)	no

Bats were detected during hibernacula monitoring from 2000–2011 in galleries of the southern Harz Mountains, Germany.

**Figure 5:**
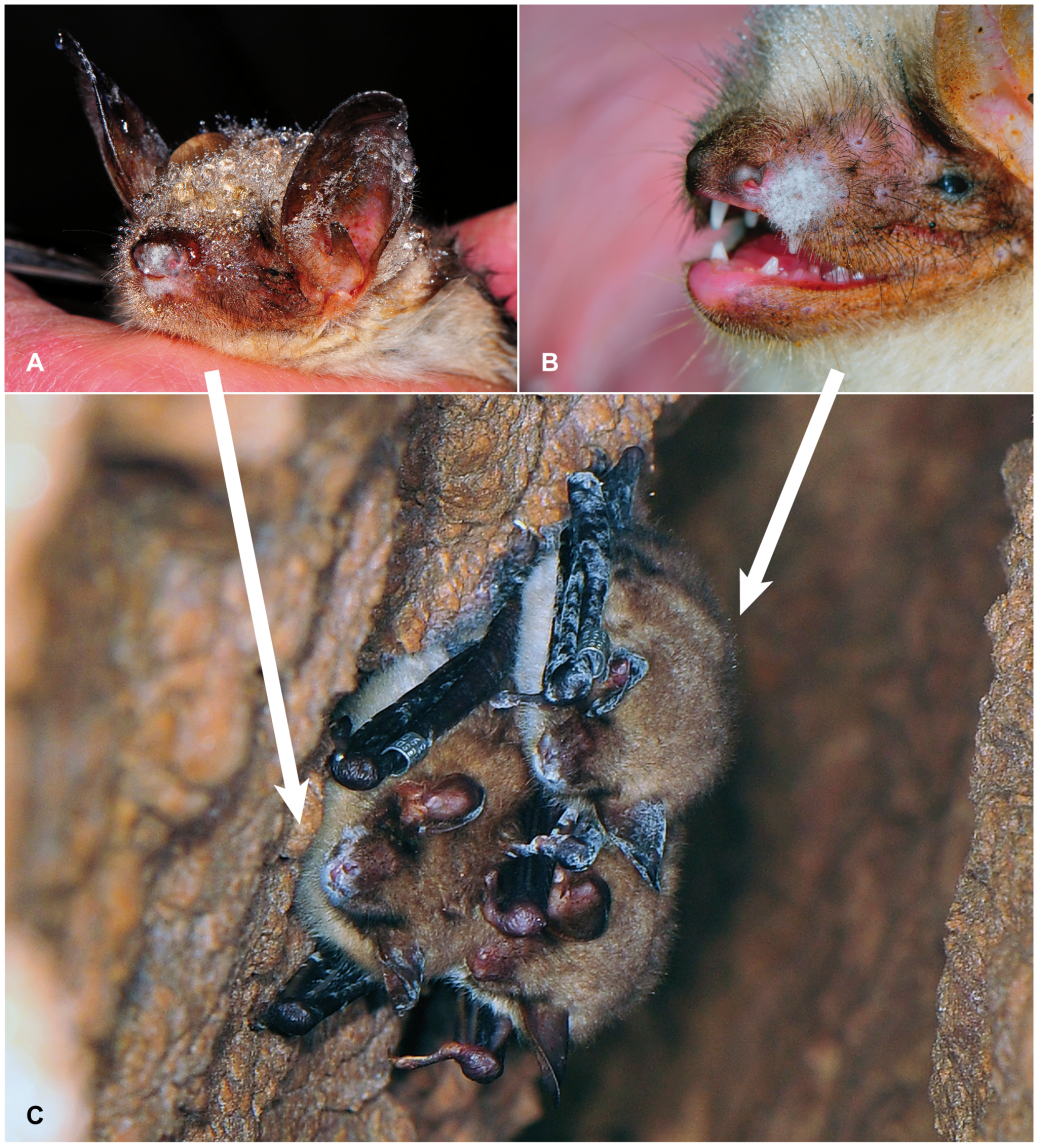
Development of *Geomyces destructans* colonization over 3 weeks’ time on naturally infected bats. 5A+B: Two banded *Myotis myotis* with mild facial fungal patches on 5^th^ February 2011. 5C: Recapture of these bats on 26^th^ February 2011 with marked fungal colonization over most of the glabrous skin parts and the snout.

In 2011, 2 banded *M. myotis* with very small patches of fungal growth on nose and ear rims were observed in a hibernaculum in Saxony on the 5^th^ February (Fig. 5A+B). Three weeks later the same animals were found again with their wing membranes, ears and muzzle markedly covered with numerous coalescent fungal patches (Fig. 5C).

## Discussion

One of the features of North American bats suffering and dying from WNS is the invasion by fungal hyphae of *G. destructans* reaching deeply into the subcutaneous tissue, causing ulcerative necrotic areas and the destruction of wing membranes [6]. The mildest microscopic changes seen in the wing membranes are cup-like epidermal erosions filled with fungal hyphae. Ulceration occurs commonly, i.e. the epidermal basal membrane is destroyed by fungal invasion spanning the full thickness of the wing membrane. When the muzzle is involved, fungal hyphae fill hair follicles, invad sebaceous and apocrine glands, and extend into the regional connective tissue obscuring epithelial boundaries of the adnexa. While typically these hibernating bats have an absence of inflammatory reaction in their skin even with extensive fungal invasion, in some animals edema and neutrophilic granulocytes as well as occasional intradermal abscesses can be observed in the regional connective tissue [6]. The severe injuries are thought to provoke increased arousal frequencies in the bats with significantly shortened torpor bouts [3]. Presumably, the ulcerated epidermal surface of the wing membranes loses its barrier function to maintain homeostasis of body fluids, thus accelerated fluid evaporation gives rise to severe dehydration and likely causes the bats to arouse [11]. This increase in arousal frequency and its associated high energy cost prematurely reduces the fat storage of the bats, inducing emaciation before the end of the hibernation period. Dehydration and emaciation then accounts for the death of the affected hibernating bats.

In contrast to North America, European hibernating bats infected with *G. destructans* are found in many countries but to date no mortalities are known to be associated with the fungus [8]. Similarly, changes in hibernation behavior such as frequent day flights in cold winter have not been observed [13]–[16]. Recently Pikula et al. [22] reported on histological investigations of 2 *M. myotis* found dead in a hibernaculum in the Czech Republic. The examination of these animals showed fungal colonization consistent with *G. destructans*. While most fungal hyphae on these 2 animals were found in the superficial skin, some hyphae were also invading the connective tissue of the dermis. Unfortunately, as the bats were found some time after their death and their organs were rendered by autolysis it was not possible to conclude the cause of death.

In the present study, wing punch biopsies of 11 hibernating live bats with macroscopic visible fungal growth on their wing membranes were collected and immediately fixed for microscopic examination. Additional skin samples were taken from one euthanized bat and were also examined by microscopy. Histologically, in this animal fungal hyphae were clearly visible in the epidermis of muzzle and wing membranes, but they were mostly restricted to the very superficial layer, i.e. stratum corneum, or the lumen of hair follicles often encasing the hair shafts. Using the histopathologic scoring system implemented by Reeder et al. [7] this bat would receive severity score 1. However, 2 other animals we investigated revealed multifocal localized intraepidermal neutrophilic infiltration associated with intralesional fungal hyphae. In their investigations on bats recovered from WNS, Meteyer et al. [6], [23] describe the presence of similar inflammatory reactions in the regional connective tissue of the skin of North American bats. In European bats such inflammatory reactions seem limited to the epidermis and do not extend beyond the epidermal basement membrane. Similarly, in the examined biopsies fungal hyphae invasion appeared restricted to the epidermis and adnexae without deep invasion into the underlying connective tissue, in contrast to the invasion of dermal connective tissue as is well documented in North American bats. Histological images of a euthanized bat from the Czech Republic with visible fungal colonization show epidermal changes similar to our findings [22]. Interestingly, even a severely compromised animal like the French bat (FR 1) described above, seemingly a prime candidate for an overwhelming fungal invasion, evidenced no ulceration of the wing membranes by connective tissue invasion of the fungus. However, although we tried to sample one of the most affected areas the limited size of the biopsy prevents conclusive decisions about the entire wing surface.

One of the morphologic structures for assessing skin diseases is the basal membrane, a dense laminar formation of collagen which separates the cell layers of the outer epidermis and its adnexae from the underlying connective tissue of the dermis. The basal membrane is regarded as one of the most important defensive barrier against injuries. Once this structure is breached, e.g. by an invading infectious agent, severe damage can result as the physiologic function of the skin, like body fluid homeostasis, is at risk. Many studies have shown that hibernating bats in North America can suffer from extensive necrosis of their wing membranes [2], [3], [6] and resulting scarring can be observed in animals surviving the infection [23], [24]. However, it was also shown that bats were able to recover and heal these lesions [23], [25]. Although the number of analyzed European samples has been very limited compared to North America, extensive skin damages with destruction of the basal membrane has not been observed in these European samples (this study, [22]), supporting the hypothesis that infection by *G. destructans* does not have the same serious consequences for bats in Europe compared to North America.

The reasons for the differences between North American and European bats are not clear, but evidence supports the hypothesis that bats in Europe might have co-evolved with the fungus while bats in North America have only been recently exposed to it [3], [8], [13], [14]. Parallel experimental infections conducted on North American *M. lucifigus* with *G. destructans* isolates from North America and Europe resulted in similar lesions, strengthening the hypothesis that *G. destructans* was introduced from Europe into North America [3]. Histological examination of the wing membrane biopsies of European bats with *G. destructans* showed that fungal hyphae remain mainly in the superficial corneal stratum. In cases where the hyphae invaded deeper layers of the epidermis, they were embraced by pustular neutrophilic infiltration. One biopsy (HU 1) seemed to represent a very early stage of such neutrophilic epidermal infiltration as well-demarcated microabscesses occurred within the epidermal layers. It could be speculated that over time, European bats might have developed an unspecific epidermal immune response, which restricts *G. destructans* colonization to the epidermis.

Regardless of these findings, multiple epidermal cup-like clusters of fungal hyphae, identical to cupping erosions described in North American bats [6], could particularly be found in the skin of the snout, where multiple hair follicles were largely expanded by densely interwoven hyphae. Such follicular hideouts could serve as a preservative source for repeated fungal growth over multiple hibernation seasons. However, Meteyer et al. [23] investigated *G. destructans* infected bats 70 days after recovery and found no evidence of remaining fungal structures on wing membranes or snout. Experiments under laboratory conditions showed that *G. destructans* samples stored on dry cotton swabs for 8 months at 24°C were not able to germinate [26], suggesting that *G. destructans* might not be able to survive on bats that are active (with temperatures >24°C) through the summer season (6–8 month).

Nothing has yet been reported about the growth rate of *G. destructans* on hibernating European bats under natural conditions. But the observation that within 3 weeks the wings and snouts of 2 banded bats from Saxony were covered with white fungus hints towards a fast growth rate under ideal conditions. Regardless of whether characteristic facial fungal patches are newly acquired or re-emerging in each hibernation season, recaptures of banded *M. myotis* with repeated detection of characteristic facial fungal patches provide evidence that European bats can survive repeated *G. destructans* infections for several years. Despite the variations in the observation dates, archival field data of annual hibernacula monitoring from southern Harz Mountains, Germany, are in accordance with other publications showing that in most years the fungus was not visible to the naked eye before mid February [13]–[15].

Differing lengths of winter and hibernation periods between North America and Europe are often discussed as a possible reason for the differing impact of *G. destructans* on the respective bat populations. In North America, WNS associated aberrant hibernation behavior as well as WNS lesions are already detectable after January [27] and recent experimental *G. destructans* infections in *M. lucifigus* confirm that WNS with deep invading fungal hyphae can be reproduced after 3 months of hibernation [3]. In our study, all bats investigated hibernated for at least 90–120 days without developing deep fungal tissue invasion. The microscopic features of superficial *G. destructans* infection in the skin of European bats could point towards a less severe fungal growth as compared to North American bats.

While it has been experimentally shown that bat to bat contact results in the transmission of *G. destructans* between the animals [2], it is unknown how common this transmission pathway is under natural conditions. In spring, immediately after their arousal, bats start to rigorously groom themselves and remove all visible fungi from their body, while some bats achieve this already in between torpor bouts during hibernation [13]. In contrast to the temperature of euthermic bats, most cavernous hibernacula have a rather constant low temperature and remaining infectious spores of *G. destructans* only need contact to a suitable substrate to start to grow. In autumn, during the swarming season when large numbers of bats aggregate to mate, they begin to enter the hibernacula and it seems likely that conidia can be swept up from cave walls by their bodies and subsequently exchanged between animals during mating [13]. The detection of *G. destructans* in caves has been shown for North America and Europe likewise [13], [28]. An alternative route of infection might take place during hibernation, when aerial hyphae on wing membranes and snouts produce innumerable conidia. Many bat species, like *M. myotis*, will occasionally move between torpor bouts and choose a different hanging place, often in close proximity to conspecifics, easily transmitting fungal conidia.

Unless European bats have evolved specific behaviors to limit Gd transmission between individuals, transmission pathways are likely to be the same in North American and European bats; however, the impact of *G. destructans* infections on bats seems to differ drastically between the continents.

## Conclusions

Numerous hibernating bats from North America succumb to infections with *G. destructans*. In contrast, in European bats even severely covered by fungal mycelia, fungal hyphae seem to be restricted to the epidermis with occasional formation of neutrophilic pustules. Invasion of the dermis and ulceration could not be found despite including several heavily infected animals. However, with the limited size of wing punch biopsies, areas of deeper invasion might have been missed. The recapture of banded bats over several years with repeated detection of fungal growth characteristic for *G. destructans* further supports the hypothesis that *G. destructans* has a minor impact on hibernating bats in Europe.
